# Reply to Zarguan et al. Comment on “Budryn et al. Hydroxybenzoic Acids as Acetylcholinesterase Inhibitors: Calorimetric and Docking Simulation Studies. *Nutrients* 2022, *14*, 2476”

**DOI:** 10.3390/nu14224860

**Published:** 2022-11-17

**Authors:** Joanna Grzelczyk, Grażyna Budryn, Horacio Pérez-Sánchez

**Affiliations:** 1 Institute of Food Technology and Analysis, Faculty of Biotechnology and Food Sciences, Lodz University of Technology, Stefanowskiego 2/22, 90-537 Lodz, Poland; 2 Structural Bioinformatics and High-Performance Computing Research Group (BIO-HPC), Computer Engineering Department, Universidad Católica de Murcia (UCAM), Guadalupe, 30107 Murcia, Spain

We thank Prof. Zarguan’s group for their comment [1] on our article [2]. We fully agree that the strategy for the screening of AChE inhibitors must begin with simple models, e.g., biochemical/biophysical or computational testing, and then, after initial satisfactory results, may be continued with in vivo models in order to respect the life of experimental animals. Biochemical or biophysical tests can be very different in nature, but they are expected to quickly and efficiently assess the binding kinetics of drug candidates, to optimize screening, and to deepen our understanding of the mechanisms underlying enzyme inhibition. One such test in our opinion is the ITC assay, in which the enzyme reacts with the substrate and the inhibitor, and the observed reaction effect is the heat change in the system. It is quite versatile, as most chemical reactions generate or consume heat. This method can be used to test reversible covalent and non-covalent enzyme inhibitors. In general, the literature on the testing of enzyme activity in the presence of inhibitors via the ITC method is extensive [3,4,5,6].

ITC-based enzyme kinetic measurements have many advantages, and the use of conventional enzyme assays to study the kinetics of inhibition may have several disadvantages, for example, experiments must be repeated many times with different concentrations of inhibitors. With the ITC technique, the inhibitor concentration changes with successive injections. It can be performed under dilute physiological solution conditions and even spectroscopically opaque conditions. The ITC is equally applicable to virtually any enzyme and does not require the development of a custom assay based on fluorogenic or colorogenic substrates or the post-reaction separation of products and substrates by chromatography or electrophoresis.

The great advantage of ITC is the possibility of using natural substrates. This is especially true when the parameters obtained for chemically modified analogs of colorogenic or fluorogenic substrates do not correspond to the parameters obtained for the native substrate via the ITC. The ITC thus provides a simple way to accurately characterize the interactions of enzymes with their biologically relevant molecular partners. Furthermore, ITC can be used for reactions that are too fast or to slow for standard methods. Unlike spectroscopic or spectrophotometric measurements, in which enzyme, substrate, and inhibitor solutions are combined before starting the measurement, the ITC measures the heat flow while the reagents are mixed. Moreover, unlike other techniques that deduce catalysis rates indirectly from substrate and product concentrations, ITC detects real-time heat flow, giving a direct reading of enzyme activity and how it changes in response to inhibitors. It is usually very difficult and sometimes nearly impossible to get this information from non-calorimetric tests. The ITC measures the rate of catalysis in a direct manner, making it an ideal sensitive method for quantifying rapid, inhibitor-dependent changes in enzyme activity. The ITC can generate enzyme kinetic parameters along with thermodynamic information from a single experiment. In a micro-ITC, protein is used at concentrations of nmol/L, and the cell volume is only 0.25 mL, so very small amounts of protein are needed.

Obviously, the more complex the model, the more variables are taken into account, and the closer it is to real conditions. Thus, we agree that drugs are best tested in clinical trials, but less sophisticated models must first be used in order to gain approval from the relevant ethics committee. In subsequent studies, we will take into account the bioavailability of phenolic acids in the Caco-2 model, as it was demonstrated in another study [7]. We want to design a food matrix from which phenolic acids will be absorbed as effectively as possible. We also aim to cooperate with an advanced research center to confirm our assumptions, including the College of Health Sciences at the International Faculty of Medicine, International University of Rabat.

## References

[1] Zarguan I., Benjouad A., Belayachi L. (2022). Comment on Budryn et al. hydroxybenzoic acids as acetylcholinesterase inhibitors: Calorimetric and docking simulation studies. *Nutrients* 2022, *14*, 2476. Nutrients.

[2] Budryn G., Majak I., Grzelczyk J., Szwajgier D., Rodríguez-Martínez A., Pérez-Sánchez H. (2022). Hydroxybenzoic acids as acetylcholinesterase inhibitors: Calorimetric and docking simulation studies. Nutrients.

[3] Abis G., Pacheco-Gómez R., Bui T.T., Conte M.R. (2019). Isothermal titration calorimetry enables rapid characterization of enzyme kinetics and inhibition for the human soluble epoxide hydrolase. Anal. Chem..

[4] Di Trani J.M., De Cesco S., O’Leary R., Plescia J., do Nascimento C.J., Moitessier N., Mittermaier A.K. (2018). Rapid measurement of inhibitor binding kinetics by isothermal titration calorimetry. Nat. Commun..

[5] Di Trani J.M., Moitessier N., Mittermaier A.K. (2018). Complete kinetic characterization of enzyme inhibition in a single isothermal titration calorimetric experiment. Anal. Chem..

[6] Wang Y., Wang G., Moitessier N., Mittermaier A.K. (2020). Enzyme kinetics by isothermal titration calorimetry: Allostery, inhibition, and dynamics. Front. Mol. Biosci..

[7] Grzelczyk J., Szwajgier D., Baranowska-Wójcik E., Budryn G., Zakłos-Szyda M., Sosnowska B. (2022). Bioaccessibility of coffee bean hydroxycinnamic acids during in vitro digestion influenced by the degree of roasting and activity of intestinal probiotic bacteria, and their activity in Caco-2 and HT29 cells. Food Chem..

